# Evaluating the Effect of a Molecular Point-of-Care Test on Acute Respiratory Infections in General Practice: Protocol for a Cluster Randomized Trial

**DOI:** 10.2196/72842

**Published:** 2025-09-08

**Authors:** Kirubakaran Balasubramaniam, Line Maria Simonsen, Line Planck Kongstad, Trine Thilsing, Sonja Wehberg, Jesper Hallas, Liza Sopina, Jesper Bo Nielsen, Gritt Overbeck, Elisabeth Assing Hvidt, Dorte Ejg Jarbøl, Tina Lein Rasmussen, Jens Søndergaard

**Affiliations:** 1 Research Unit of General Practice Department of Public Health University of Southern Denmark Odense M Denmark; 2 DaCHE - Danish Centre for Health Economics Department of Public Health University of Southern Denmark Odense Denmark; 3 Clinical Pharmacology, Pharmacy and Environmental Medicine Department of Public Health University of Southern Denmark Odense Denmark; 4 Section of General Practice Department of Public Health University of Copenhagen Copenhagen Denmark

**Keywords:** point-of-care, acute respiratory infections, general practice

## Abstract

**Background:**

Acute respiratory infections (ARIs) are frequent reasons for medical consultations in general practice and can lead to unnecessary recontacts. Introducing new point-of-care (POC) polymerase chain reaction (PCR) diagnostic equipment may offer an attractive and efficient way of providing a more precise and exact microbial diagnosis. Successful uptake of POC PCR equipment could potentially lead to a reduction in recontacts with benefits for both staff and patients. However, introducing new diagnostic technology is a complex intervention and several contextual factors may impact the implementation.

**Objective:**

This study aims to evaluate the effect of POC PCR test availability in general practice on the subsequent (1) number of recontacts to the general practitioner (GP) for patients with symptoms of ARIs (primary outcome) and (2) hospital admissions, deaths, antibiotic prescriptions, health-related quality of life, GP and patient satisfaction, costs, cost-effectiveness, and contextual facilitators and barriers conditioning the implementation process (secondary outcomes).

**Methods:**

This study is a cluster-randomized, crossover, nonblinded superiority trial with a 1:1 allocation ratio between usual care (control) and POC PCR test availability (intervention). Questionnaire data are collected at day 0, 7, 14, and 28 after the initial contact (health-related quality of life, absenteeism and presentism among patients, and patient satisfaction) and after finalization of the study period (GP satisfaction). Data on recontacts, hospital admissions, redeemed antibiotic prescriptions, costs, and deaths will be retrieved from the Danish national registries. The implementation process will be evaluated based on data from interviews with users of POC PCR tests (i.e., GPs, staff, and patients) and from observations in the clinics in line with Medical Research Council guidelines.

**Results:**

As per the randomized crossover design carried out during September 2023 to March 2024 investigating a sample of 100 GP clinics, we expect to obtain an in-depth and multifaceted understanding of the effects of the availability of the POC PCR test equipment in general practice.

**Conclusions:**

This study will provide valuable information about the diagnostic conditions and possibilities in general practice and provide insights into the organization of primary health care.

**Trial Registration:**

ClinicalTrials.gov NCT06120153; https://clinicaltrials.gov/study/NCT06120153

**International Registered Report Identifier (IRRID):**

DERR1-10.2196/72842

## Introduction

### Acute Respiratory Infections, Recontacts, and Antibiotic Prescriptions

A very common reason for visiting Danish general practice is symptoms of acute respiratory infections (ARIs), and diagnostic accuracy is important to ensure appropriate treatment [1]. Danish general practitioners (GPs) handle approximately 98% of these cases without referring the patients to further diagnosis or treatment in hospitals [2]. It is important for the quality of care that patients are diagnosed as fast as possible, and that the GP and the patient are confident about the diagnostic accuracy. If not, the patient may be unnecessarily worried and contact health services again. Recontacting health services is sometimes appropriate, for example, if the patient’s symptoms worsen. Unnecessary recontacts are, however, unsatisfactory to both the patient and the clinician as a high number of recontacts indicate diagnostic and treatment deficits and a lack of reassurance of the patient. In addition, recontacts and hospital admissions are costly for society. For these reasons, the study investigates the following research question: “Do patients with ARIs diagnosed in general practice with access to point-of-care (POC) polymerase chain reaction (PCR) equipment benefit in terms of reducing recontacts to general practice in a 1-week period after the initial contact?”

In addition to investigating recontacts as the primary outcome, the study also allows for insights into patterns related to antibiotic prescription. In Denmark, around 75% of all antibiotics are prescribed in general practice, and ARIs are some of the most common reasons for prescriptions [3,4]. Even though Denmark is among the countries with the lowest consumption of antibiotics worldwide, there is still room for improvement in the total use of antibiotics and in the choice of regime, that is, broad spectrum versus narrow spectrum antibiotics [5]. Antibiotic overuse may lead to microbial resistance, and if the overall consumption is not restrained, infections with resistant bacteria will be a major problem for both the individual patient and the health care system. Hence, initiatives to significantly reduce the prescription and use of antibiotics are highly warranted [6]. Whether symptoms arise from bacteria or from a viral infection is often uncertain in the primary care setting [7], and when precise diagnostics are desirable, it often takes 1 to 3 working days and requires sending test samples to external laboratories. Due to diagnostic uncertainty, GPs may in some situations prescribe antibiotics even in cases where the patient’s symptoms are caused by a viral infection. Introducing POC PCR diagnostic equipment in general practice may improve diagnostic accuracy, and thereby reduce revisits as well as prescription and use of antibiotics.

Introducing practice changing diagnostic tools into primary care extends beyond the mere adoption of a new diagnostic technology; it is a complex intervention that involves significant interactions among health care professionals, technology, and the health care system itself. Complex interventions are characterized by multiple components that interact within their implementation context [8]. These include the behaviors of health care providers and patients, the environment in which the intervention is deployed, and the outcomes it aims to influence. The complexity of integrating, for example, POC PCR testing into daily practice is particularly evident in the multifaceted processes it entails, from conducting the test and interpreting its results to making informed clinical decisions and adapting the health care infrastructure to support its use.

The use of rapid PCR testing for respiratory tract infections has been extensively examined in hospital and emergency department settings [9]. The effects on clinical and resource related outcomes vary significantly between studies [10-12]. A recent systematic review and meta-analysis found no association between rapid PCR testing and antibiotics use, return visits, length of stay, or hospitalization among patients with ARI in emergency departments [13]. The clinical effects of POC PCR testing of patients with ARI in general practice have not been extensively examined. However, a feasibility study conducted during the COVID-19 pandemic showed promising results in terms of feasibility and clinical utility [14,15].

This paper describes a protocol for a cluster randomized controlled trial focusing on the effects of introducing POC PCR testing (Cobas LIAT) for ARIs in general practice in Denmark. To qualify the study protocol, 2 preparatory studies were conducted, demonstrating barriers and facilitators for introducing POC PCR technology in general practice, including user experiences from the clinicians [16,17].

### Objectives

The study is organized in 3 work packages (WPs) with the following objectives.

#### WP1: Effectiveness of POC PCR Availability for ARI in GP Clinics

The following are objectives 1 to 6: to evaluate the effectiveness of POC PCR availability in general practice on (1) the number of recontacts to the GP for patients with symptoms of ARIs (primary outcome); (2) the number of redeemed antibiotic prescriptions, (3) health-related quality of life, and (4) GP and patient satisfaction. In addition, (5) the number of hospital admissions and (6) deaths are included as safety measures.

#### WP2: Cost-Effectiveness Associated With POC PCR Availability in GP Clinics

The following are objectives 7 and 8: to evaluate (7) the costs and (8) the cost-effectiveness of POC PCR availability in general practice compared with usual care.

#### WP3: Process Evaluation of Introducing and Implementing POC PCR Availability in GP Clinics

The following is objective 9: to identify and analyze contextual facilitators and barriers as well as behavioral factors that influence potential uptake of the implementation process tied to the POC PCR equipment.

This study uses recontacts as a primary outcome of evaluating the effectiveness of the test, as this is a less-explored phenomenon associated with ARIs. Denmark is already one of the countries with the lowest consumption of antibiotics, which is why this measurement is also included in the study (as a secondary outcome in the analyses). While investigating patterns tied to antibiotic prescriptions are important, the scope of this study is to explore the wider operations of a general practice setting, which a primary but not isolated focus on recontacts allows.

## Methods

### Study Setting

The study is conducted in 4 Danish regions (Region of Southern Denmark, Region Zealand, North Denmark Region, and Central Region of Denmark), comprising a general population of approximately 4 million individuals and 2340 GPs [18].

In Denmark, GPs receive reimbursement from the administrative regions for all consultations and tests included in the collective agreement between the PLO and the regions. The role of the regions in this study is to create and administer project specific reimbursement codes that will enable calculations of the number of patients with ARIs and the number of POC PCR tests performed. All Danish GPs are accustomed to register reimbursement codes in their electronic patient record system after each consultation, and all codes are linked to the patient’s personal identification number.

### Recruitment and Eligibility Criteria

GP clinics are eligible for participation if they are situated in one of the participating regions and receive reimbursements from the administrative regions. GP clinics are invited by letters with written information about the project. The letters are sent from the clinical trial unit at the University of Southern Denmark (SDU), who also conducts follow-up phone calls and distributes additional material if requested by the clinic. Interested GP clinics will sign a collaboration agreement with SDU and receive additional information. Patients are eligible for participation if they contact a participating GP clinic with symptoms of ARI during the study period. All clinics and patients taking part in the effect study and the economic evaluation (WP1 and WP2) will receive information about the project and data collection according to the Danish Data Protection Act Section 10. Specifically, as the studies of WP1 and WP2 are conducted on register data, no patients will be identifiable, and thus, no consent is needed. The qualitative process evaluation (WP3) necessitates oral and written information, and all patients are asked to sign an informed consent form. All participation is voluntary, and every patient and professional is informed that they can revoke their consent at any point should they regret their participation.

### Allocation

The study is designed as a cluster randomized crossover study with the GP clinic as the unit of randomization, that is, all GPs affiliated to a GP clinic are randomized to either intervention (A) in the first period and usual care (B) in the second (AB sequence) or in the BA sequence. Allocation of the general practice clinic is randomized using a computer algorithm and allocation is 1:1 between the AB or BA sequence. When an eligible clinic commits to the trial, the project statistician informs the project management about the next allocation in the allocation sequence and the clinic is randomized according to this, that is, blinded randomization allocation is used.

Randomization at the patient level is not considered feasible, as an ongoing randomization procedure in the clinic would be time consuming and pose a risk of bias and interference with the diagnostic procedure and subsequent decision-making by the GP.

Therefore, randomization is stratified by type of clinic, that is, partnership- or single-physician clinics. Large collaborative clinics are randomized as one unit in the group of non–single-physician clinics. A block randomization in blocks of 10 is used.

### Intervention

#### Pretrial Information and Training

**Figure 1:**
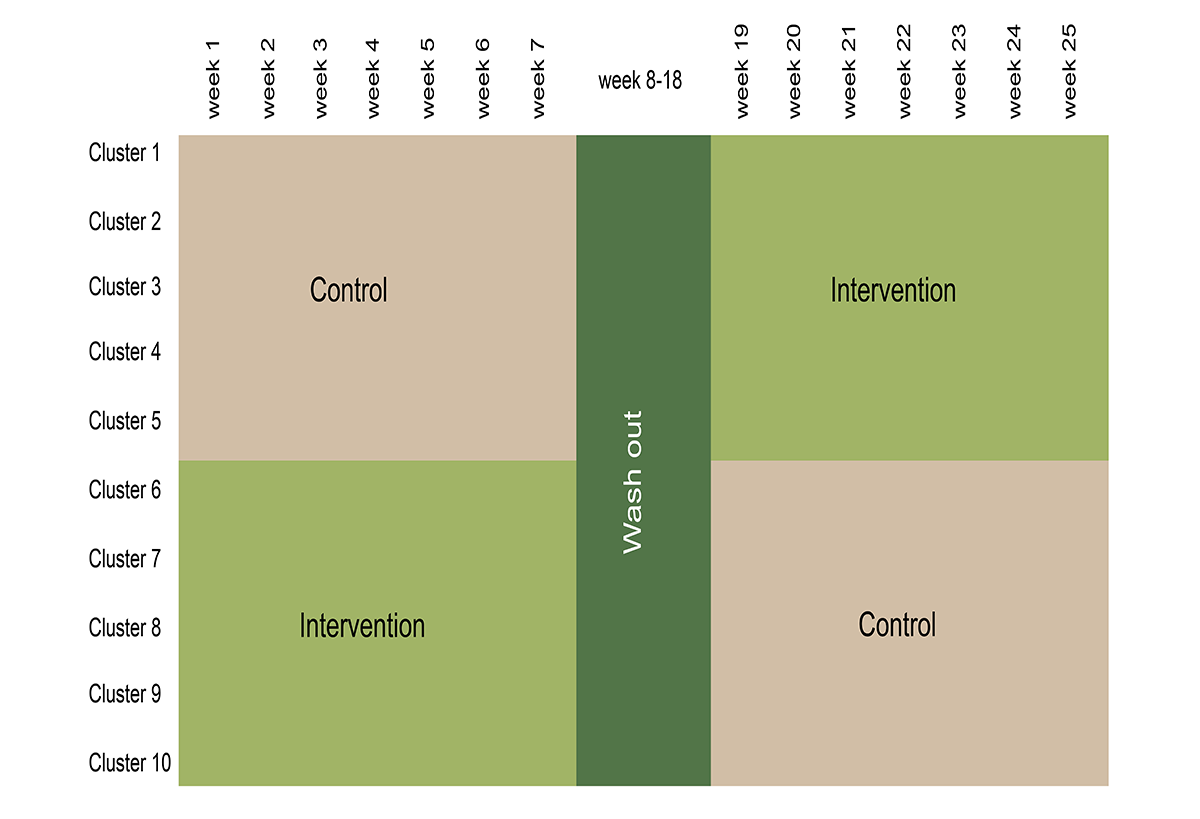
Illustrates the phases in the randomized controlled trial’s control and intervention periods as well as the washout period. In the washout period, Roche removes, cleans, and reinstalls the equipment from the intervention group, so it is ready for the control group for the final phase of the trial.

The study will be conducted over a period of 25 weeks (Figure 1).

In the month leading up to the intervention period, the POC PCR device will be installed in the intervention clinics by staff from Roche Diagnostics A/S, and assays and equipment necessary for the testing will be sent to the clinics free of charge. Just before entering the trial period, each clinic will be trained by staff from the clinical trial unit from SDU. During the training session, the clinic will receive further information about patient inclusion, registration of eligible patients in the reimbursement system, and whether the clinic is about to enter an intervention period, including training on how to perform the POC PCR tests. In addition, all clinics will receive an information poster for patients to display in the waiting area. All GP clinics will receive a fee of DKK 6000 (approximately €800 or US $840; 1DKK= US $0.14) for participating in the study. In addition, GPs will be remunerated for performing the test (DKK 71.48 [approximately €9.50 or US $10] per test equivalent to already established POC tests). The clinics participating in the process evaluation will receive another DKK 1000 per interview (approximately €134), and DKK 2000 (approximately €268 or US $280) per whole day during observation studies.

An overview of the pretrial activities is presented in Textbox 1.

**Pretrial instructions and training during intervention and control periods.**

**Intervention period**

1. Introductory instructions about the trial are provided for the clinics (how to include and register patients with acute respiratory infection [ARI])
2. Training in the use of Cobas LIAT respiratory testing portfolio (group A Strep, Influenza A/B, SARS-CoV-2, and respiratory syncytial virus)
3. Instruction to include all patients contacting the clinic with symptoms of ARI during a 7-wk period and possible point-of-care testing, if deemed relevant

**Control period**

1. Introductory instructions about the trial are provided to the clinics (how to include and register patients with ARI)
2. Instruction to include all patients contacting the clinic with symptoms of ARIs during a 7-wk period for treatment as usual.

#### Activities During the Study Period

All patients with ARI contacting the participating GP clinics during the intervention or control period will be included in the study. Before or after the consultation with the GP, the clinic staff will hand out information materials about the project, including a leaflet with a QR code and a link to a questionnaire.

In the control period, patients with ARI will receive usual care, usually comprising no or limited diagnostic tests at the clinic, or nasal or pharyngeal swap for testing at an external laboratory with an expected response time of 1 to 3 days. During the intervention period, the POC PCR equipment will be used when the GP deems it relevant.

Developed by Roche Diagnostics A/S, the POC PCR test system tested in this study comprises an analysis instrument and assay tubes for testing (1) StrepA, (2) Sars-Cov-2 and Influenza, and (3) Influenza or respiratory syncytial virus (RSV). The test is performed on oropharyngeal (StrepA), nasopharyngeal (SARS-Cov-2 or Influenza, and Influenza or RSV), or nasal (Influenza or RSV) swap samples. Running the test takes approximately 20 minutes per assay.

After the consultation, the GP registers the project specific reimbursement codes in the electronic patients record system. Three different reimbursement codes are used: the first code is for the registration of each individual patient with ARI contacting the clinic. This code is used in both control and intervention periods. The second code is for each time the POC PCR test is performed (allowed only once per patient per consultation even though it is possible to perform multiple tests within the same consultation), and the third code is for situations where the test result is conveyed by phone or email to the patient the day after the test is performed. The last two codes are for intervention periods only. GPs are only remunerated for performing the test.

### Statistical Analysis and Sample Size Calculations

#### Sample Size Calculation

Outcomes are on the individual patient level, and the clustering involves the entire GP clinic, not the individual GP. A balanced design with a common total cluster size and common period effect was adopted. As the study is a superiority trial, results from the feasibility study [16] and Danish national register data will inform the choice of parameters as inputs to the sample size formula derived by Li et al [19]. The intended effect size was fixed at a reduction from 0.32 to 0.28 based on the register results, which approximately corresponds to the distance between the median or mean to the 25th percentile. With a significance level of 0.05, power at 0.8, within-period within-cluster correlation of 0.02, and across-period within-cluster correlation as 0.01 to detect a difference between a prevalence of 0.32 recontacts in the control group and 0.28 in the intervention group, the sample size was calculated as 108 clinics, contributing 50 contacts each in the trial period. Pragmatically, the inclusion of 100 clinics was chosen.

#### Blinding (Masking)

Due to the intervention procedure (an oropharyngeal, nasopharyngeal, or nasal swab) and the use of project specific reimbursement codes, it is not possible to blind the patients and GPs.

#### Outcomes

The primary outcome is modeled as group difference in number of patients with ARI recontacting the GP from the date of the initial contact (day 0) and the following 7 days in the intervention and control periods [19]. Secondary outcomes include safety outcomes such as group difference in the total number of redeemed antibiotic prescriptions (day 0 and following 7 days), the total number of hospital admissions, and deaths (from the date of the initial contact [day 0] and following 14 days) and total treatment costs, productivity costs, quality-adjusted life years, and incremental cost-effectiveness or utility ratio (from day 0-28) [14]. In addition, patient (day 0) and GP satisfaction (after the end of trial) is evaluated along with patients’ health-related quality of life (day 7, 14, and 28). Furthermore, the study elucidates several aspects and factors based on the process evaluations’ qualitative design, which allows us to assess the quantitative outcomes. Where the quantitative outcomes emphasize measured effects, the qualitative design allows insight into, for instance, the participants’ perceptions of diagnostic practices in the clinic, the emergence and coordination of behavior, experiences, and interactions with the device, and thus explains how people use and perceive the device the way they do [16]. Multimedia Appendix 1 describes the different outcomes and points of interest including information on the specific measure, metric, applied instrument, time period, data source, and target population in a table overview.

### Data Collection

#### A Triangulated Design

The evaluation is based on both quantitative and qualitative methods. Quantitative data will be collected from national administrative registries and via web-based questionnaires; qualitative data will be collected via interviews and observations, including video observation (see details in subsequent sections).

#### Questionnaire Data

The GPs or staff will distribute a leaflet with a QR code (and a link) to a web-based survey to all patients with ARI aged ≥15 years. For patients aged <15 years, the caregiver will be asked to answer the questionnaire. In the first questionnaire, the personal identification number and contact information will be collected, and the subsequent questionnaires will be distributed via a link in email and SMS text message at day 7, 14, and 28. In case of nonresponse, reminders will be sent within 2 days. Participants who complete all 4 questionnaires will be entered into a lottery for 1 gift card of Dkr 1000 for completing the first questionnaire and 1 gift card of Dkr 2500 for completing all 4 questionnaires. Questionnaires will be managed via a secure electronic questionnaire system (SurveyXact). Each questionnaire will take approximately 5 to 10 minutes to complete. Furthermore, a questionnaire will be sent to all participating GP clinics at the end of the trial followed by a reminder after 2 weeks in case of no response. The questionnaire will take approximately 5 minutes to complete. In it, the GPs will be asked about their overall experience of the equipment associated with (1) support for performing precise diagnostics, (2) support for clinical judgment, (3) the influence of the test results related to clinical management of patients, (4) the influence of the test results in patient communication concerning their condition, (5) disruption of consultation, (6) disruption in overall workflow, and (7) whether the equipment belongs in general practice. In addition, there is space for the GPs to write comments.

#### Register Data

Comprehensive public register data on contacts and services provided in primary care, prescription of antibiotics, and hospital contacts and various characteristics of the GP clinics will be retrieved from the Danish Health Data Authority [20]. Furthermore, information on morbidity, socioeconomic status, and various demographic characteristics on each patient will be obtained from Statistics Denmark [21]. Data from the different registers are linked by the patients’ personal identification number. No data will be retrieved from the registers until the protocol paper is accepted for publication.

#### Qualitative Data

Qualitative data will be collected through an ethnographic fieldwork, which includes several key components. First, 10 pre- and postintervention interviews will be conducted with GPs and staff across 11 clinics. In addition, participant observations will take place in 5 clinics both before and during the intervention along with video observations in 3 out of the 5 clinics. Furthermore, 14 patient interviews will be held after the intervention.

Interviews with health professionals will focus on value assessments and sense-making processes tied to their experience with diagnostic technologies in general and the POC PCR equipment specifically. Participant observation will be conducted (with and without video-recorded participation) to obtain in-depth knowledge about everyday working life; organizational and communicative behavior; and interactions between health professionals, staff, and patients with (and without) the POC PCR equipment in the GP clinic. Finally, patient interviews will focus on their experience with use of the POC PCR equipment, feelings of diagnostic certainty, and reflections concerning the perceived impact of the POC PCR test on relational and communicative processes.

#### Data Management

Questionnaire data will be collected by SurveyXact and stored on secure servers at University of Southern Denmark. After data cleaning, questionnaire data will be transferred to secure scientific servers at Statistics Denmark and linked to register data via the personal identification number, after which all data will be pseudonymized.

Qualitative data will be collected by means of digital recorders during interviews, by field notes during observations, and using a GoPro video camera (handheld by the researcher) during video observation. After collecting data, they will be stored on secure servers provided by SDU. NVivo software (QSR International) will be used for transcriptions and analytical purposes.

After publication of the study, the data manager will ensure that all study data are deleted.

### Data Analysis

#### Effectiveness of GP Access to POC PCR

Demographic characteristics of the included patients and GP clinics will be presented. Analysis of the binary primary outcome uses a standard logistic regression model on the individual (contact) level, accounting for clustering of patients in clinics by generalized estimating equations. Primary analysis will follow an intention-to-treat approach, and we want to show superiority. A corresponding analysis strategy will be applied for hospital admissions, deaths, and redeemed antibiotic prescriptions. A level of 5% will be set as statistically significant. Analyses will be performed with the most recent version of Stata (StataCorp).

#### Within-Trial Cost-Effectiveness

The cost-effectiveness analyses will take both a national health care perspective and, separately for the purpose of sensitivity analysis, a limited restricted societal perspective, including health care costs, patient costs (travel cost), and productivity costs for patients or their caregiver (in case the patient is a child aged <15 y). Cost-effectiveness will be reported in terms of the incremental cost-effectiveness ratio (ICER) expressed as the difference in costs divided by the difference in outcome, between patients in the intervention and control groups. Costs will be estimated from the time of initial contact (day 0) and over a follow-up period of 28 days and calculated by category. Due to the short duration of the study, costs and outcomes will not be discounted. Costs will be expressed in Euros, in 2024 conversion rates. Effect will be measured as the total number of recontacts within 7 days and quality-adjusted life years within 28 days. Quality-adjusted life years are calculated as the area under the utility curve for each patient based on patients’ health-related quality of life measured by EQ-5D-5L for patients aged >15 years (in Denmark, individuals become responsible for managing their own health care at the age of 15, therefore not a requiring parental intermediary or proxy completion of the EuroQoL 5-Dimension) and valued using Danish value sets [22,23]. Missing costs or quality-of-life data will be handled by multiple imputations. Differences in mean costs and effect will be estimated using separate generalized linear regression models, with appropriate distribution and link functions for costs and outcomes. The models will control for treatment, time point, treatment-by-period interaction as fixed effects, and cluster at the GP level as a random effect. Furthermore, the models will adjust for baseline health care cost (6 months before the initial contact) and baseline utility [24]. Nonparametric confidence intervals for costs, effect, and ICER will be computed using a 2-stage bootstrapping technique with 1000 replications [25] and the 2.5th and the 97.5th percentile of the 1000 bootstrap replications. Conventional methods for estimating uncertainty around the ICER, such as cost-effectiveness acceptability curves, will be applied as well. Sensitivity analyses will be conducted to test assumptions, uncertainty around variable estimates, and the robustness of results.

#### Qualitative Process Evaluation of Introducing and Implementing POC PCR in GP Clinics

All qualitative analyses will be analyzed using the reflexive thematic analysis by Braun and Clarke [26]. Following this approach, the transcripts will be initially read and reread, with participants taking notes along the way using the annotations feature in CASDAQ software NVivo (version 14; QSR International). Afterward, still using NVivo, the data will be coded inductively. Codes are then grouped into themes and subthemes that capture, on a general level, the identified factors promoting and impeding potential users’ use of the POC PCR equipment.

Specifically, preintervention interviews with GPs and staff focus on the organization of the clinic and everyday experiences in the clinic as well as expectations regarding the upcoming intervention, diagnostic processes, and technology.

Postintervention interviews with GPs and staff focus on experiences of the usefulness, effect, and meaningfulness of the POC PCR test during the intervention.

Interviews with patients focus on the participants’ experience with their symptoms, diagnosis, treatment, and use of health technologies for diagnostic purposes and also on their reflections concerning the perceived impact of the POC PCR test on relational and communicative processes (eg, whether the test enhances feelings of trust, of being taken seriously, and of reassurance). Participants accompanying a child aged <15 years will be interviewed relative to their considerations and experiences associated with the experience of being in the consultation with the child.

Analysis of the preintervention video observation data will focus on the learning processes in connection with training sessions and characteristics of standard practices in the clinic relating to patients with ARI. Analysis of video observation data collected during intervention will focus on how usefulness and potential implementation barriers and facilitators play out during the everyday working life of GPs and staff.

### Ethics and Dissemination

#### Research Ethics Approval

As the project entails an evaluation study of an already CE-certified diagnostic tool, approval from the Danish Medicine Agency and the Scientific Committees of Medical Ethics (from Danish: De Videnskabsetiske Medicinske Komiteer) has been waived. The study has been assessed and approved by Local Ethics Committee at University of Southern Denmark (case number 23/1584) and University of Southern Denmark’s Research and Innovation Organization (SDU RIO; journal number: 11.907).

In addition, the project has been registered on ClinicalTrials.gov (NCT06120153).

#### Consent or Assent

Informed consent is not required for collection of questionnaires and register-based data according to the Danish Data Protection Act Section 10. For the interviews and observations, written and oral consent is obtained from the GP or staff before video observation in the clinic. When a patient arrives for consultation, oral and written information about the project is given and consent for observation is obtained. Only if the patient consents, the consultation is observed and recorded. After observation, the researcher asks the patient for permission to a follow-up interview. The patient is provided a copy of the consent form and written information about the project, General Data Protection Regulation, and contact information for the project team.

None of the questionnaires or planned interviews contain psychological trigger questions nor is the intervention expected to give rise to any unwanted effects, as the POC PCR equipment used applied is a CE-certified diagnostic tool already in clinical use in other settings. Whether the POC PCR test is used by the health professional is entirely up to the individual to decide. Whether the GPs or patients choose to participate in the project does not have any influence on their treatment, and it is completely voluntary to fill out questionnaires or participate in interview- and observation studies.

#### Confidentiality

Data from interviews and observations will be stored at a secure server provided by SDU. Personal information (names, cities, and other locations) will be pseudonymized during the stages of transcribing the data and rewriting the field notes. For the purpose of scientific publications, screenshots from video recordings will be deidentified as sketches, as drawings, or with blurred features.

#### Access to Data

In line with the collaboration agreement signed between SDU and Roche Diagnostics A/S, only researchers and project team members from SDU have access to the data.

#### Dissemination Policy

In accordance with the contractual agreement signed by SDU and Roche Diagnostics A/S, SDU can present and publish data and results from the project regardless of the outcome in concordance with Danish law. Full disclosure is given to Roche Diagnostics A/S related to the sponsor’s supporting role in the project in all written and oral presentations. Roche Diagnostics A/S has the right to review and offer comments for written or oral presentations as well as the right to intervene if any confidential information provided by Roche Diagnostics A/S is made public. Around 30 days before submission for publication, Roche Diagnostics A/S must receive the material intended for publication or public presentation for review. However, it is SDU’s right to decide whether any comments or inputs from Roche Diagnostics A/S will be included in the publications. Roche Diagnostics A/S is allowed to use publications or abstracts from the project when reference is made to the author or authors in accordance with applicable law, and only after such publications have been made publicly available.

## Results

As per the randomized crossover design carried out during September 2023 to March 2024 investigating a sample of 100 GP clinics, it is expected to get in-depth and multifaceted understanding of the effects of the availability of the POC PCR test equipment in general practice.

Specifically, in terms of effectiveness, costs, and how organizational and experiential factors may work as barriers and facilitators in the uptake of the intervention in the clinic, both quantitative generalizable results and qualitative explanations of contextual conditions are expected.

## Discussion

### Principal Findings

Swift and precise diagnosis of ARIs in general practice is of great importance for obtaining appropriate health care use and use of antibiotics as well as both patient and GP satisfaction. This cluster randomized clinical trial is designed to explore the effects of availability of POC PCR in general practice. However, the pragmatic design comes with certain limitations.

### Strengths and Limitations

First, there is limited possibility to ensure that the patient receives the postcard and fills out the questionnaire, which may hamper studying the patients’ quality of life and satisfaction. Second, the quality of the quantitative data is highly dependent on GPs applying the project specific reimbursements codes to all relevant patients. As coding after each patient contact is everyday routine for Danish GPs, the coding practice itself is not a new task for the participating clinics. However, coding difficulties associated with the new reimbursement codes cannot be ruled out, which may in turn lead to a slight underreporting. Finally, while the POC PCR test equipment allows for conducting 5 ARI tests, the current test portfolio does not allow testing for, for example, pertussis and mycoplasma. As such, it could influence both the professional and patient experience and perception of what is considered quality and precise diagnostics and, by the mere introduction of increased testing that the POC PCR equipment promotes, the introduction of more testing could lead to an overemphasis of testing for the sake of testing. Moreover, it takes 20 minutes to run a test and a consultation in a Danish general practice usually is reimbursed for only 10 minutes. This practical factor may also influence the uptake of the intervention and the experience of the usefulness of the technology, it may even create additional workload in the clinic.

Despite the possible limitations, the described project will explore the potential effects of use of the POC diagnostic equipment in general practice. A major strength of the study is the study design, mirroring real-world practice, where clinicians themselves are in charge of deciding which diagnostic approaches are warranted, rather than forcing clinicians to use tools that they do not deem relevant. Furthermore, including clinics of different sizes across the country ensures a variety of clinics in the sample, which also reflects real-world circumstances. Another strength of this study is the use of register data, which provides unambiguous linkage of trial participants to high-quality, nation-wide registers. These registers allow analyses of both socioeconomic data and data from the health care systems in the secondary care setting. Moreover, the triangulated design of the qualitative process evaluation that combines interviews with observations of health professional and patient behavior in the natural setting of the clinics allows for the attainment of in-depth knowledge of how the intervention works in practice, how the professionals may perform workarounds, and how it can be adjusted around daily life. In addition, insights into how they reflect on their own actions and what they deem and perceive as barriers and facilitators for the possible implementation of the new technology in the future is obtained. While it can be expected to be an added workload to manage the discrepancies of the test’s running time versus time for consultations, it is not uncommon that the GP finds tools meaningful as they are embedded in practice, if the gain from using it adds an overall value. Furthermore, patient interviews will illuminate the patients’ needs, expectations, and experiences of diagnostic practices, technology, and the patients’ relations to the clinic and staff, which allows for understanding and explaining reconsulting behavior and more.

### Conclusions

In conclusion, the study gives the possibility to explore the potential effect of not only the availability of the POC PCR equipment tied to recontacts (WP1) but also the economic consequences (WP2) and the organizational factors as well as professional and patient-related experiences tied to the implementation of the diagnostic equipment (WP3).

## Appendix

Multimedia Appendix 1 Study objectives and their associated measurements, specific metrics, possible use of instruments, data collection period, evaluation points, data sources, and target groups.

## Abbreviations

ARI: acute respiratory infection
GP: general practitioner
ICER: incremental cost-effectiveness ratio
PCR: polymerase chain reaction
POC: point-of-care
RSV: respiratory syncytial virus
WP: work packages
