# Supplementary material for: Evaluating the Effect of a Molecular Point-of-Care Test on Acute Respiratory Infections in General Practice: Protocol for a Cluster Randomized Trial
Source: JMIR Res Protoc. 2025 Sep 8;14:e72842. doi: 10.2196/72842 (PMC12455145; doi:10.2196/72842)
Supplement: Multimedia Appendix 1 [file resprot_v14i1e72842_app1.docx]

**Table S1**: The table below operationalizes the study’s objectives and their associated measurements, specific metrics, possible use of instruments, data collection period, evaluation points, data sources, and target groups.

| **Objectives** | **Primary outcome** | **Definition of measure** | **Specific metric** | **Instrument** | **Data collection period** | **Evaluation point** | **Data source** | **Target group** |
| --- | --- | --- | --- | --- | --- | --- | --- | --- |
| 1. | Re-contacts to General Practitioners | Total number of patients re-contacting the GP within 7 days from the initial contact.  The recontacts  include any daytime contacts to general practice. | Intervention period compared to control period – recontact (Y/N) | - | Day 0-7 | Evaluated on day 7 | Register data | Acute respiratory infection (ARI) patients |
|  | **Secondary outcomes** |  |  |  |  |  |  |  |
| 2. | Hospital admissions | Total number of infections related hospital admissions within 2 weeks from the initial contact. | Intervention period compared to control period – admission (Y/N) | - | Day 0-14 | Evaluated on day 14 | Register data | ARI patients |
| 3. | Deaths | Total number of deaths within 2 weeks from the initial contact. | Intervention period compared to control period – death (Y/N) | - | Day 0-14 | Evaluated on day 14 | Register data | ARI patients |
| 4. | Antibiotic prescriptions | Total number of redeemed antibiotic prescriptions within 7 days from the initial contact. | Intervention period compared to control period  - redeemed prescription (Y/N) | - | Day 0-7 | Evaluated on day 7 | Register data | ARI patients |
| 5. | Health-related quality of life | Health related quality of life measured in five dimensions: mobility, self-care, usual activities, pain/discomfort, and anxiety/depression. | Change from baseline, compared to patients treated in control GP clinics | EuroQol-5L Dimension questionnaire (EQ-5D) | day 0, 7, 14 and 28 | Evaluated on day 7, 14 and 28. | Questionnaire data | ARI patients age >14 |
| 6a. | GP satisfaction | Satisfaction measured on a five-point likert scale, ranging from “strongly disagree” to “strongly agree” | Intervention period compared to control period | - | After end of the trial | Evaluated at the end of the trial | Questionnaire data | GPs |
| 6b. | Patient satisfaction | Satisfaction measured on a five-point likert scale, ranging from “strongly disagree” to “strongly agree” | Intervention period compared to control period | - | Day 0 | Evaluated on day 0. | Questionnaire data | ARI patients and caregivers |
| 7a. | Total treatment costs | Intervention costs, health care utilization and travel costs | Difference in costs between patients in intervention and control period | - | Day 0-28 | Evaluated on day 28. | Register data | ARI patients |
| 7b. | Productivity costs | Costs related to hours missed from work, impairment while at work, and impairment in regular activities. | Difference in costs between patients (caregivers for patients >14 years) in the intervention and control period. | Adjusted version of Work  Productivity and Activity Impairment General Health (WPAI-GH). | Day 0,7,14 and 28. | Evaluated on day 28. | Questionnaire data | ARI patients and caregivers |
| 8a. | Cost-effectiveness | Incremental cost-effectiveness ratio | Difference in costs divided by differences in recontacts |  |  | Evaluated on day 28. | Questionnaire data and register data | ARI patients |
| 8b. | Cost-utility | Incremental cost-effectiveness ratio | Differences in costs divided by differences in Quality-adjusted-life-years (QALYs) | EQ-5D-5L (Danish value sets) |  | Evaluated on day 28. | Questionnaire data and register data | ARI patients age >14 |
| 9. | Evaluating implementation process | Thematizing interactions with the equipment as well as experiences and perceptions of diagnostic practices in the clinic among GPs, staff and patients. | Facilitators and barriers regarding implementation, and conditions associated with the device’s usefulness | Pre- and post-interview guides  Semi-structured interview guide  Observation studies | Pre-, during and post intervention | Evaluated pre, during and post intervention. | Interview and observation data | GPs and staff and ARI patients- and caregivers |
